# *E. coli* Nissle 1917 modulates host glucose metabolism without directly acting on glucose

**DOI:** 10.1038/s41598-021-02431-8

**Published:** 2021-12-01

**Authors:** Theodore A. Chavkin, Loc-Duyen Pham, Aleksandar Kostic

**Affiliations:** 1 Section on Pathophysiology and Molecular Pharmacology, Joslin Diabetes Center, Boston, MA USA; 2 Department of Microbiology, Harvard Medical School, Boston, MA USA

**Keywords:** Microbiome, Diabetes, Applied microbiology

## Abstract

Managing postprandial glycemic response, or the increase in blood sugar following a meal, is a crucial component to maintaining healthy blood sugar in patients with diabetes. To test whether oral probiotics can impact postprandial glycemic response, *E. coli* Nissle 1917 (EcN) was evaluated in an oral glucose tolerance test. Oral gavage of EcN concurrent with a glucose bolus reduced the post-gavage glycemic response in mice. However, there was no difference in glycemic response when comparing EcN to a mutant deficient in glucose metabolism. This suggests that while EcN can alter glycemic response to a glucose bolus, this effect is not mediated by direct uptake of glucose. Of the possible indirect effects EcN could have, gastric emptying rate was highlighted as a likely cause, but EcN had no effect on gastric emptying rate in mice. This leaves many more possible indirect explanations for the interaction between EcN and host glucose metabolism to be explored in future work.

## Introduction

One of the key aspects of maintaining healthy blood sugar in patients with diabetes is managing the postprandial glycemic response^1–3^. Shortly after a carbohydrate rich meal, free sugars reach the small intestine where they are rapidly absorbed into the intestinal lumen and into the bloodstream^4,5^. Rising blood sugar levels cause a glucose stimulated insulin secretion (GSIS), stimulating glucose uptake in peripheral tissue^6^. However, in patients with diabetes, GSIS is impaired, either by reduced function of the insulin producing pancreatic beta islets (as in type I diabetes and MODY genetic diabetes), or by reduced sensitivity of peripheral tissue to insulin signals (as in type II diabetes)^7,8^. This leads to impaired postprandial glycemic response, causing long periods of elevated blood sugar after meals, and often requiring pharmaceutical intervention to manage^9^.

The study of the human gut microbiome has uncovered many promising targets for “functional probiotics”' with experimentally verified effects. For example, *Akkermansia muciniphila* has been targeted as an anti-obesogenic microbe, with supporting data in mice and in clinical trials^10–12^. *Bacteroides thetaiotaomicron* has received similar attention as a potential probiotic for reducing weight gain in a high fat diet context^13–15^. And recently our group has identified *Veillonella atypica* as a candidate for an endurance-enhancing microbe in the context of exercise^16^. Microbes are a potentially powerful tool for impacting health, as they exist naturally in the gut at high abundance, have biological connections to a massive number of diseases and biological processes, and can be isolated, cultured, and formulated for diet supplementation^17^.

In the context of altering postprandial glycemic response, we hypothesized a clear mechanism by which the microbiome could impact glucose uptake and improve glycemic profiles. Many bacteria have high affinity for glucose, and in the gut microbiome, bacteria could feasibly compete with the host for glucose absorption and degradation. To combat this, the sites of carbohydrate degradation and glucose uptake, the stomach and upper small intestine, are harsh environments with low colonization rates due to high acidity, fast lumen flow rate, antimicrobial compounds, and a loose mucus layer that impairs adhesion^18,19^. We hypothesized that by boosting the local concentration of glucose-degrading bacteria in the stomach and small intestine, the microbiome could compete with the host for glucose uptake, reducing the amount of glucose that makes it to the bloodstream. To this end, we chose *Escherichia coli* Nissle 1917 , a probiotic strain that is used commercially as a probiotic and is closely related to model strains of *E. coli* that are well characterized for their fast growth rates, high affinity for glucose, and relative ease of engineering^20^.

In this study, we propose that oral supplementation of probiotic bacteria with high affinity for glucose could impact glycemic response to an oral glucose bolus. We show that concurrent administration of probiotic *E. coli* Nissle 1917 with a glucose bolus causes a reduction in the glycemic response. We then tested whether *E. coli* glucose consumption is necessary to see this change in glycemic response but found that a glucose-null mutant had no effect when compared to wild type. We then hypothesized that bacterially-induced changes in gastric emptying rate could explain the change in glycemic response, however we observed no change in gastric emptying in *E. coli* Nissle supplemented mice versus PBS control. This suggests that while *E. coli* Nissle appears able to modify the glycemic response, the mechanism is complex. Importantly, we demonstrate that the intuitive explanation of simple bacterial absorption of dietary glucose is likely not responsible for this effect.

## Results

**Figure 1:**
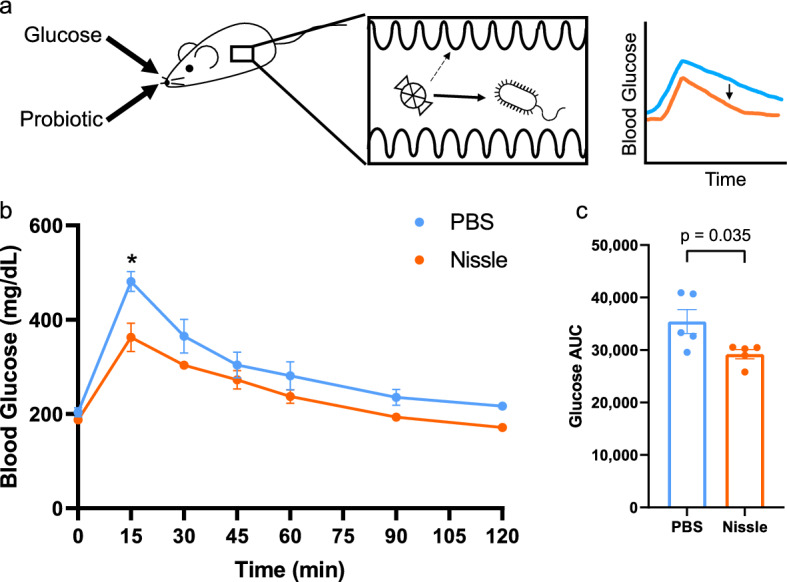
*E. coli* Nissle 1917 improves glycemic response in healthy mice. (**a**) Experimental design. (**b**) Oral glucose tolerance test (OGTT) showing blood glucose over time following oral glucose gavage. Points represent mean and bars represent SEM (n = 5). (**c**) Area under the curve (AUC, in mg/dL ∗ s) of **b** (n = 5). Points represent individual mice and bars represent mean and SEM. p values calculated in (**b**) using repeated measures ANOVA with Tukey’s post-hoc test, and in (**c**) using student’s T-test.

We first set out to test the hypothesis that the glucose metabolic capacity of live probiotics can impact glucose uptake rate in the gastrointestinal tract (Fig. 1a). 8-week-old C57-BL/6J mice were gavaged with 10^10^ CFU of *E. coli* Nissle 1917, concurrently with 2 mg/kg glucose, and blood glucose was monitored over 2 h (Fig. 1b). At each time point measured, Nissle-treated mice showed a trend of lower average blood glucose than vehicle control, though this difference was not significant. However, the area under the blood glucose curve (AUC) was significantly lower in Nissle-treated mice compared to vehicle control (Fig. 1c). We then explored whether other probiotic bacteria could elicit a similar response. However, administration of a cocktail of probiotic Lactobacillus strains showed no impact on average blood glucose at any time point measured nor the average AUC (Supplementary Fig. S1). These data suggest that while probiotic bacteria can impact the glycemic response to a bolus of glucose, this response is species specific, prompting further investigation into what causes *E. coli* Nissle specifically to have this effect.

**Figure 2:**
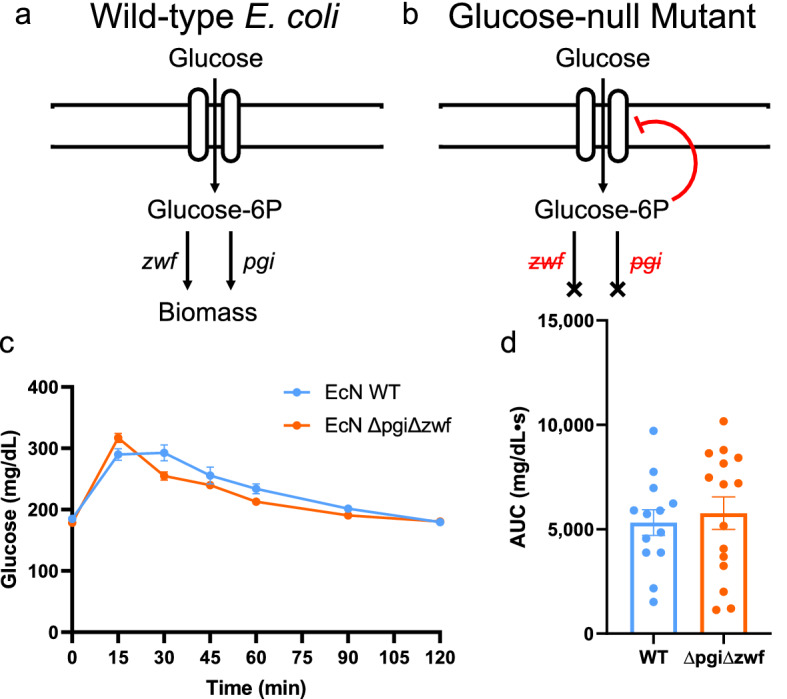
A glucose-null mutant of *E. coli* Nissle 1917 has no impact on blood glucose AUC compared to wild type. (**a**) Simplified view of glucose metabolism in *E. coli*. Hexokinase transports glucose from extracellular space into the cytoplasm and phosphorylates, generating glucose-6-phosphate. Glucose-6P is processed via *zwf* into the pentose phosphate pathway or via *pgi* into glycolysis. Knocking out both genes causes buildup of glucose-6P, eliminating further glucose uptake via feedback inhibition. (**b**) OGTT showing blood glucose over time following oral glucose gavage (n = 14). Points represent mean and bars represent SEM (**c**) AUC of the OGTT (n = 14). Points represent individual mice and bars represent mean and SEM. p values calculated in (**b**) using repeated measures ANOVA with Tukey’s post-hoc test, and in **c** using student’s T-test.

*Escherichia coli* strains are well characterized for their high rate of glucose uptake. To test whether glucose uptake was necessary for the improvement of glycemic response, we first generated a glucose-null mutant of *E. coli* Nissle. *E. coli* metabolizes glucose via central carbon metabolism, primarily through glycolysis and the pentose phosphate pathway (Fig. 2a). Hexose transporters take up glucose and phsophorylate to glucose-6-phosphate, which is incorporated into glycolysis via phosphoglucoisomerase (*pgi*), or into the pentose phosphate pathway via glucose-6-phosphate dehydrogenase (G6PDH, encoded by gene *zwf*)^21^. In *E. coli* strains lacking both *pgi* and *zwf*, glucose-6-phosphate accumulates in the cell, inhibiting hexokinase transporters via feedback inhibition. Using *E. coli* Nissle 1917 as a base strain, we generated the double knockout EcN ∆pgi∆zwf and confirmed that it was unable to grow in minimal media with glucose as the sole carbon source, while growth on glycerol was unaffected (Supplementary Fig. S2). In an OGTT, mice supplemented with EcN ∆pgi∆zwf showed no significant change in glycemic profile compared to wild type EcN, and the AUC was unchanged as well (Fig. 2b,c). Thus, while EcN appears to affect the glycemic profile in OGTT, this effect is not dependent on EcN possessing functional glucose metabolism, suggesting that this effect is dependent on some other property of the bacteria.

**Figure 3:**
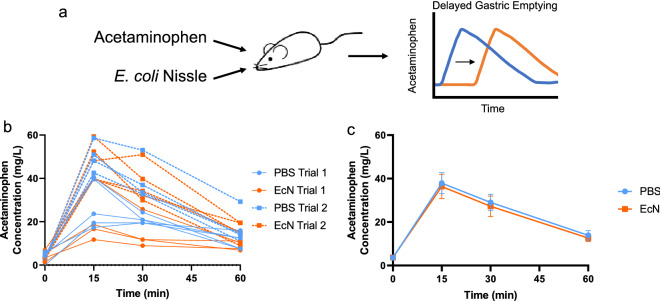
Impact of *E. coli* Nissle on gastric emptying rate. (**a**) Experimental design. Acetaminophen is co-administered with bacteria or PBS as an oral gavage. Acetaminophen acts as a tracer to monitor rate of gastric emptying and further clearance from the blood. (**b,c**) Concentration of acetaminophen in the blood over time following oral gavage (n = 9). In (**b**), points represent individual mice, while in **c** points represent mean and bars represent SEM. p values calculated in **b** and **c** using repeated measures ANOVA with Tukey’s post-hoc test.

For *E. coli* Nissle to impact glycemic response without directly interacting with glucose, it would have to have some effect on host physiology, impacting glycemic response indirectly. A prime candidate for this indirect effect is gastric emptying, as microbiota and their small molecule effectors have been shown to impact gastric emptying rate, and gastric emptying rate has a direct effect on glucose absorption rate^22^. To test whether Nissle impacts gastric emptying rate, we used acetaminophen as a tracer, co-administering a bolus of acetaminophen with either Nissle or PBS as an oral gavage. By monitoring concentration of acetaminophen in the blood, gastric emptying rate can be estimated as the rate of influx of acetaminophen into the bloodstream from the stomach (Fig. 3a). However, we observed no clear effect of Nissle on gastric emptying rate. Inter-mouse variability was high, as was the batch effect between the two trials conducted (Fig. 3b). Aggregated, we observed no difference in average blood concentration of acetaminophen at any time point measured (Fig. 3c). These data suggest that Nissle has no observable impact on gastric emptying rate in mice, and thus gastric emptying cannot explain our initial observation.

## Discussion

In this study, we proposed the hypothesis that *E. coli* Nissle could act as a glucose “sponge” by competing with the host for glucose uptake. Promisingly, we observed that Nissle reduced the glycemic response in mice compared to PBS vehicle control. However, we acknowledged that PBS was not an adequate control to conclude that this effect was caused by direct competition. To test this further, we generated a glucose-null mutant of Nissle (EcN ∆pgi∆zwf), which is unable to grow on or metabolize glucose. However, in a mouse OGTT, EcN ∆pgi∆zwf showed no change in glycemic response compared to wild type, meaning that functional glucose metabolism was not necessary for the glycemic impact we initially observed. Furthermore, this observation meant that in absence of a direct effect, the next best explanation for our initial findings would be an indirect effect: bacterial gavage impacting an aspect of host physiology which alters glycemic response in a separate downstream response. We then tested one likely candidate for this indirect effect via gastric emptying rate, however we found that *E. coli* Nissle had no effect on gastric emptying.

Eliminating this possibility does little to whittle down the long list of potential bioprocesses a probiotic microbe could be interacting with that alter glycemic response. Microbiome signals have been shown to affect the secretion of several endocrine hormones, including GLP-1 and GIP^22^ which are both involved in postprandial insulin secretion^23^. Other affected signals include PYY, which has a more complicated role in glucose homeostasis^24^, and amylin, which affects blood glucose via delaying gastric emptying^25^. Microbiome signals have also been shown to interact with the neurological system, via the gut-brain axis^26^. Neurological connections to glucose sensing and homeostasis represent another possible explanation^27^. The microbiome has also been shown to interact with metabolism directly via small molecule secretion. Microbiome derived succinate absorbed from the intestinal lumen can induce intestinal gluconeogenesis, which improves glucose homeostasis and reduces glycemic response^28^. There is even emerging research suggesting that there may be a resident pancreatic microbiome, and oral supplementation of *E. coli* specifically has been observed entering the pancreatic ducts at high concentrations^29^. Direct pancreatic stimulation could affect glucose homeostasis via a unique mechanism, making this avenue particularly hazy and unknown. We are left without a clear explanation for our initial finding and a wide expanse of possible avenues towards an explanation which lies outside the scope of this study.

The key finding of this work is the observation that probiotic bacteria given just prior to a glucose bolus can impact the body’s response to the glucose. Our initial assumption was that this effect was caused by direct competition for glucose, as this was the most likely and most exciting possibility. However, our further experimentation showed that competition could not explain the initial observation, demonstrating that the microbiome and microbe-host interactions are much more complicated than they appear.

## Methods

### Bacterial strains and growth

To generate material for gavage, bacterial strains were grown in LB at 37 °C overnight, then subinoculated into LB at 1:100 dilution and incubated at 37 °C until OD_600_ 0.4–0.8. Cultures were then pelleted at 4000 g, 4 °C, and washed once in 1 × PBS. Pellets were resuspended in PBS to a final concentration of ~ 5 × 10^10^ CFU/mL and flash frozen in liquid nitrogen. Aliquots were stored at -80 °C. CFU/mL was determined by plating a dilution series of the aliquots onto LB agar plates and counting colonies after 24 h incubation at 37 °C.

To generate the glucose-null mutant, we utilized the lambda red recombineering system encoded on plasmid pREDTKI^30^. Deletion cassettes were designed for ∆pgi and ∆zwf by PCR amplifying FRT-CatR-FRT (chloramphenicol resistance gene) from pKD3 with 60 bp homology to the upstream and downstream regions of each gene. EcN cultures were made electrocompetent by growing cultures to OD_600_ 0.4–0.6 and washing with 10% glycerol twice and resuspending in 1/100th the original volume. Electrocompetent cultures were transformed with 10 ng plasmid DNA or 100 ng linear DNA at 3 kV 2 mm gap with 1 h recovery in SOC media before plating on LB agar plates. EcN pREDTKI cells were grown in the presence of 0.1% arabinose and electrotransformed with the ∆pgi deletion cassette, then plated on LB Chlor. Activation of the FRT recombinase at 42 °C removed the CatR gene, then the process was repeated for the ∆zwf deletion. Deletions were confirmed by sequencing across the gene and glucose-null growth phenotype confirmed by streaking overnight LB cultures on M9 minimal media with glucose or glycerol as the sole carbon source.

### Animal procedures

Protocols for mouse experiments were reviewed and approved by the Joslin Diabetes Center Animal Care and Use Committee (Protocol #2016-05). The study was carried out in accordance with the ARRIVE guidelines. All experiments were performed according the relevant guidelines and recommendations. All mouse experiments were performed using 8–12 week old male C57/B6J mice ordered from Jackson Labs. Male mice were chosen to eliminate sex variation, as well as to keep in line with most comparable studies. To reduce confounding effects, mice were distributed between groups from different litters and cages. No individuals were removed from downstream data analysis post hoc.

### Oral glucose tolerance test

For OGTT, standard best practice guidelines were followed^31^. Mice were individually caged and fasted for 5 h prior to gavage. Roughly 1 h prior to gavage, bacterial aliquots were thawed on ice. Roughly 15 min before gavage, baseline blood glucose was measured. Immediately before gavage, 670µL of 50% w/v glucose was added to 1 mL bacteria maintained at ~ 10^10^ CFU/mL and gavaged at 10µL per gram body weight. This gives final dosages of 2 g/kg BW glucose and 10^9^ CFU/g BW bacteria. Gavages were timed exactly 1 min apart, then blood glucose was measured via tail vein bleed and a handheld glucose meter at 15, 30, 45, 60, 90, and 120 min post-gavage. Area under the curve was calculated using the trapezoid rule with 0 mg/dL as baseline. For probiotic blend gavage, a commercial probiotic blend was obtained, consisting of 10^9^ CFU per capsule of *Bifidobacterium bifidum, Bifidobacterium breve, Bifidobacterium longum, Lb. acidophilus, Lb. casei, Lb. paracasei, Lb. rhamnosus, and Streptococcus thermophilus.* (CVS) This probiotic was suspended in PBS to a concentration of 10^10^ CFU/mL, as measured by CFU plating. Probiotic blend was administered identically to the frozen bacterial aliquots. For all mouse experiments, the administering researcher was blinded to the aliquot contents.

### Gastric emptying

Gastric emptying rate was measured as previously described^32,33^. Briefly, mice were fasted overnight for 20 h. Gavage mixture was prepared by adding equal volumes 2% w/v acetaminophen and either PBS or bacteria maintained at 10^10^ CFU/mL, thawed on ice as above. Baseline blood samples were collected via tail vein bleed, and mice were gavaged 250µL gavage mixture, giving final dosage of 2.5 mg acetaminophen and ~ 10^9^ CFU bacteria per animal. Blood samples were collected at 15, 30, and 60 min into heparinized tubes and stored on ice. Blood samples were centrifuged at 4 °C for 20 min at 2000×*g*. Plasma was collected and stored in separate tubes at − 20 °C. Acetaminophen was quantified in the plasma using an enzymatic colorimetric assay specific to acetaminophen (Cambridge Life Sciences).

### Statistics

Figures 1b, 2b, 3b,c.

Data were analyzed using repeated measures ANOVA with Tukey’s post-hoc test in Prism 11.0. Significance threshold set at p = 0.05.

Figures 1c, 2c.

Data were analyzed using student’s T test in Excel. Specifically, two-sample unequal variance two-tailed T-test was used. Significance threshold set at p = 0.05.

## Supplementary Information

Supplementary Information.
