# Supplementary material for: E. coli Nissle 1917 modulates host glucose metabolism without directly acting on glucose
Source: Sci Rep. 2021 Dec 1;11:23230. doi: 10.1038/s41598-021-02431-8 (PMC8636602; doi:10.1038/s41598-021-02431-8)
Supplement: Supplementary file 1 — Supplementary Information. [file 41598_2021_2431_MOESM1_ESM.pdf]

Supplemental Material for:

E. coli Nissle 1917 Modulates Host Glucose Metabolism Without Directly Acting on Glucose

Theodore A. Chavkin[1,2], Loc-Duyen Pham[1,2], Aleksandar Kostic[1,2]*

[1]Section on Pathophysiology and Molecular Pharmacology, Joslin Diabetes Center, Boston, MA, USA
[2]Department of Microbiology, Harvard Medical School, Boston, MA, USA
*Corresponding Author

Supplementary Figures

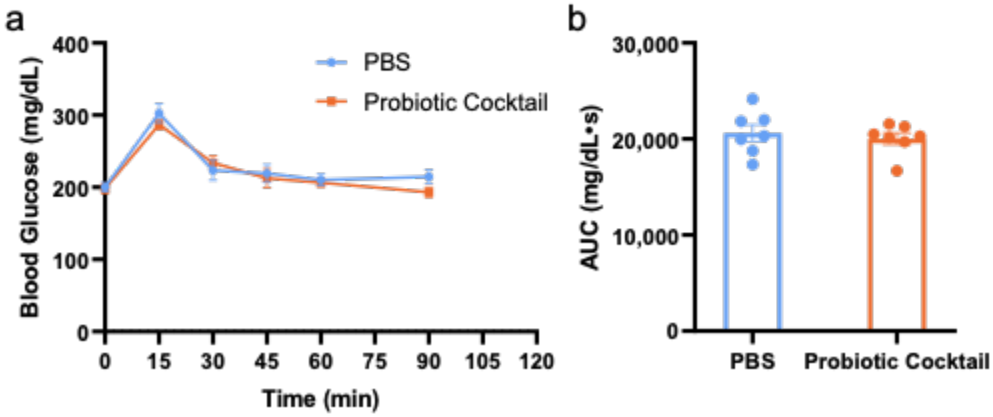

Supplementary Figure 1. A mixture of probiotic species does not impact OGTT in mice. **a** Blood glucose over time following a co-administered bolus of 1g/kg glucose and 10^9 CFU of freeze-dried probiotic mixture. **b** Area under the curve of **a**.

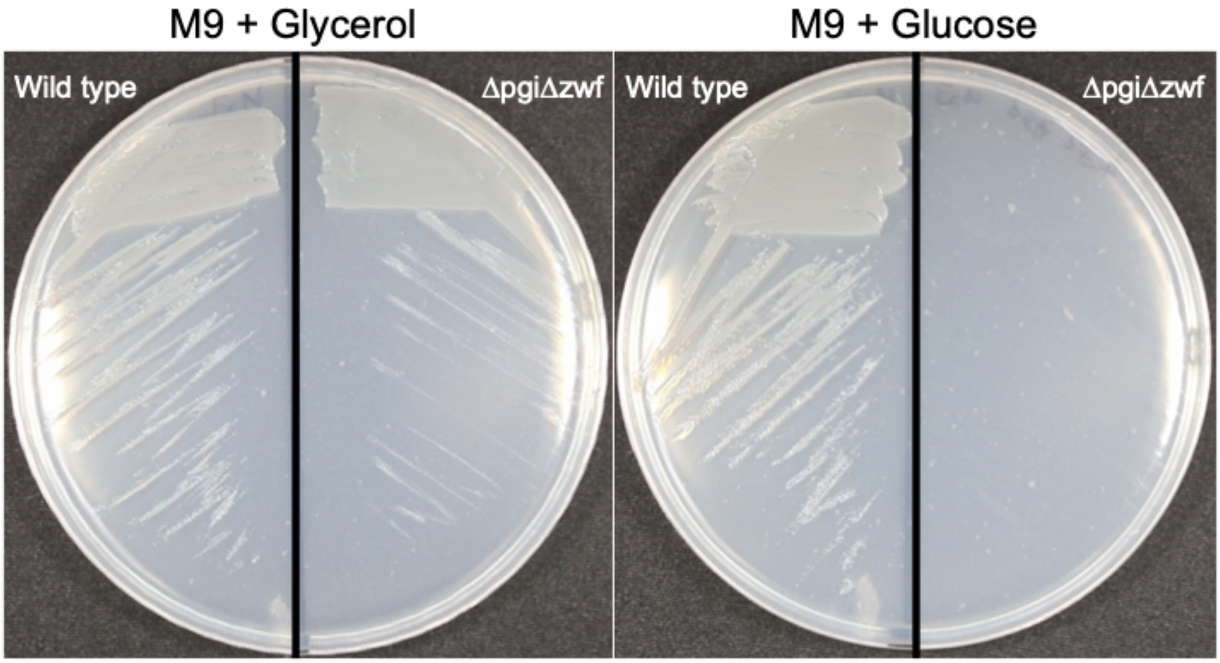

Supplementary Figure 2. EcN ΔpgiΔzwf is incapable of growth in minimal media with glucose as the sole carbon source.
